# Trading off Iodine and Radiation Dose in Coronary Computed Tomography

**DOI:** 10.3390/jcdd12050195

**Published:** 2025-05-20

**Authors:** Guillaume Fahrni, Thomas Saliba, Damien Racine, Marianna Gulizia, Georgios Tzimas, Chiara Pozzessere, David C. Rotzinger

**Affiliations:** 1 Department of Diagnostic and Interventional Radiology, Lausanne University Hospital, University of Lausanne, Rue du Bugnon 46, 1011 Lausanne, Switzerland; thomas.saliba@chuv.ch (T.S.); marianna.gulizia@chuv.ch (M.G.); chiara.pozzessere@chuv.ch (C.P.); 2 Institute of Radiation Physics, Lausanne University Hospital, University of Lausanne, Rue du Grand-Pré 1 46, 1007 Lausanne, Switzerland; damien.racine@chuv.ch; 3 Department of Cardiology, Lausanne University Hospital, University of Lausanne, Rue du Bugnon 46, 1011 Lausanne, Switzerland; georgios.tzimas@chuv.ch

**Keywords:** coronary artery disease, coronary CT angiography, radiation dosage, iodinated contrast media, contrast-induced acute kidney injury, patient-specific protocols

## Abstract

Coronary CT angiography (CCTA) has seen steady progress since its inception, becoming a key player in the non-invasive assessment of coronary artery disease (CAD). Advancements in CT technology, including iterative and deep-learning-based reconstruction, wide-area detectors, and dual-source systems, have helped mitigate early limitations, such as high radiation doses, motion artifacts, high iodine load, and non-diagnostic image quality. However, the adjustments between ionizing radiation and iodinated contrast material (CM) volumes remain a critical concern, especially due to the increasing use of CCTA in various indications. This review explores the balance between radiation and CM volumes, emphasizing patient-specific protocol optimization to improve diagnostic accuracy while minimizing risks. Radiation dose reduction strategies, such as low tube voltage protocols, prospective ECG-gating, and modern reconstruction algorithms, have significantly decreased radiation exposure, with some studies achieving sub-millisievert doses. Similarly, CM volume optimization, including adjustments in strategies for calculating CM volume, iodine concentration, and flow protocols, plays a role in managing risks such as contrast-associated acute kidney injury, particularly in patients with renal impairment. Emerging technologies, such as photon-counting CT and deep-learning reconstruction, promise further improvements in dose efficiency and image quality. This review summarizes current evidence, highlights the benefits and limitations of dose control approaches, and provides practical recommendations for practitioners. By tailoring protocols to patient characteristics, such as age, renal function, and body habitus, clinicians can achieve an optimal trade-off between diagnostic accuracy and patient safety, ensuring optimal operation of CT systems in clinical practice.

## 1. Introduction

Imaging the coronary arteries, tortuous, millimetric structures, prone to motion artifacts caused by the constant beating of the heart, represents one of the most challenging tasks in clinical radiology. Achieving high-resolution, motion-free images requires advanced imaging techniques, precise timing, and often the use of specialized equipment to overcome these challenges and ensure accurate diagnosis and treatment planning. Since coronary CT angiography (CCTA) was first reported in 1979 [1], both technology and our understanding of coronary physiology have improved dramatically [2,3,4]. In its early days, CCTA suffered from considerable limitations, hampering its application in clinical routine. Those limitations included low temporal resolution and the absence of motion correction algorithms, leading to motion artifact, limited current, and tube overheating, resulting in noisy images, especially before the advent of advanced reconstruction algorithms and the overestimation of coronary stenosis due to limited spatial resolution [5], among others. Historically, due to the limited coverage of detectors, the scanning mode used to often be helical, delivering staggering dose levels that were a real concern at the time. On top of that, the non-negligible likelihood of non-diagnostic studies due to noise or motion artifacts precluded widespread uptake. Over time, CCTA has evolved into one of the most widely used non-invasive imaging techniques for visualizing the coronary arteries and assessing the presence of coronary artery disease (CAD). CCTA is now recognized as a Class I, Level of Evidence A recommendation for the evaluation of both acute and chronic coronary syndromes, according to the recent American College of Cardiology/American Heart Association (ACC/AHA) Chest Pain Guidelines [6]. In alignment with the ACC/AHA, the 2024 ESC Guidelines also strongly endorse coronary CCTA as a first-line diagnostic tool in patients with stable chest pain and suspected obstructive coronary artery disease. CCTA receives a Class I, Level of Evidence B recommendation for patients with a low to intermediate clinical likelihood of CAD. It is preferred for its high negative predictive value and non-invasive nature. The ESC further highlights CCTA’s utility in assessing coronary anomalies, atherosclerosis burden, and plaque morphology. For acute coronary syndromes, CCTA may be considered when high-sensitivity troponin and ECG are inconclusive [7]. The use of CCTA is particularly advocated in symptomatic patients presenting with stable chest pain and an intermediate to high pre-test probability of obstructive coronary artery disease, as well as in patients with acute chest pain who are deemed to be at intermediate risk. By providing anatomical details of both the lumen and arterial wall, CCTA has established itself as a key player for CAD assessment, quantifying the severity of stenosis, evaluating plaque burden, providing risk stratification, and guiding subsequent management decisions. Today, it is well-established that the risk–benefit balance between the (much lower) radiation dose and clinical value of the diagnostic test, when applied to the right patient population, clearly favors the use of CCTA [8,9,10]. Despite technological advancements in dose reduction, real-world challenges remain, with significant variability in dose practices across centers. Patient-specific considerations, such as body habitus, are critical for tailoring imaging protocols that achieve optimal dose reduction while maintaining high image quality [11].

With state-of-the-art CT systems, radiation dose can be kept at reasonable levels while delivering valuable clinical information. To save radiation dose, it is typically advised to use heart rate control, lower tube potential (80–100 peak tube voltage (kVp)), tube current modulation, fast gantry rotation, wide-area detectors or dual-source technology, and advanced reconstruction algorithms, i.e., iterative or deep-learning-based reconstruction [12,13]. Different strategies to optimize radiation dose in CCTA are now well-established and thoroughly described in several studies [14,15,16,17]. Interestingly, contrast medium (CM) injection protocols can substantially impact the necessary radiation dose and generally receive less attention in the literature [18,19]. Since the early 1920s, CM safety has been improving steadily, leading to more limited toxicity, improved tolerability, and better contrast. However, adverse reactions still occur in 1–3% of procedures, indicating that the use of CM cannot be regarded as entirely risk-free [20]. Environmental and economic concerns about iodine use also underscore the need for careful protocol optimization [21].

Trading off radiation and iodinated contrast dose is a growing concern due to the increasing use of CCTA as a first-line diagnostic tool, concerns about radiation-induced cancer risks, and the potential for contrast-associated acute kidney injury (CA-AKI), particularly in patients with renal impairment or other comorbidities. This trade-off directly influences clinical decision-making, requiring patient-specific protocols that account for factors such as age, renal function, and body habitus. For example, younger patients may need more attention regarding radiation reduction due to the higher susceptibility and long-term cancer risks, while patients with impaired renal function may require lower iodine doses to avoid CA-AKI, even if it may necessitate higher radiation exposure. Additionally, this balance is relevant in follow-up scans for patients requiring serial imaging, where cumulative radiation exposure must be carefully managed.

This review aims to provide an in-depth analysis of the trade-off between ionizing radiation dose and iodinated contrast material dose in CCTA and to summarize the tools necessary to optimize the balance between diagnostic accuracy and patient safety in CCTA. By synthesizing the existing literature, this review will explore the benefits and drawbacks of different approaches to dose control, highlight emerging technologies, and provide recommendations for practitioners.

## 2. Technical Principles in CCTA

### 2.1. Computed Tomography System and Parameters

To obtain diagnostic images of the coronary tree, CT systems with at least 64 detector rows with a nominal thickness of ≤0.625 mm and ECG-gating are mandatory to maximize spatial resolution and limit motion artifacts (see Section 5.2) [10]. The radiation dose received by the patient during a CCTA examination depends on various factors, including the CT system, scan coverage, tube voltage, tube current, gantry revolution speed, and the individual patient’s size. In general, because of the coronary arteries’ small size and tortuous nature, both high spatial and contrast resolution are required, and both depend on noise, especially with iterative reconstruction [22]. Higher radiation doses are often inevitable for achieving low-noise images, but fortunately, recent technological developments have helped decrease the radiation dose burden in CCTA, especially with reduced tube voltage [23]. Furthermore, high temporal resolution (enabled by fast gantry rotation) and sufficient frame rate are critical to visualize dynamic coronary behaviors, such as systolic bending, intramural functional occlusion, and myocardial bridging. These phenomena, which occur transiently during specific cardiac phases, may be missed or misinterpreted if motion artifacts dominate [24,25].

Regarding CM injection, a power injector capable of delivering at least a 4 mL/s flow rate is required. Applying lower kVp settings helps enhance contrast, as it increases the attenuation of iodinated contrast agents, resulting in clearer visualization of the coronary arteries and improved diagnostic accuracy [23]. Additionally, advanced reconstruction algorithms, including deep-learning reconstruction (DLR), can further enhance image quality by reducing noise while preserving fine details, enabling the use of even lower kVp settings, and improving image clarity. However, the application of low kVp is limited by patient characteristics, as patients with a high body mass index (BMI), for example, may require an increase in kVp to ensure adequate image quality and sufficient X-ray beam penetration [26]. Furthermore, spectral CT, particularly photon-counting CT (PCCT), offers major advantages in cardiac imaging by providing enhanced contrast resolution through the use of low-energy virtual monochromatic reconstruction. Altogether, these advancements allow for high-quality cardiac imaging with reduced radiation exposure, enhancing both diagnostic precision and patient safety.

### 2.2. Coronary Lumen Attenuation

**Figure 1:**
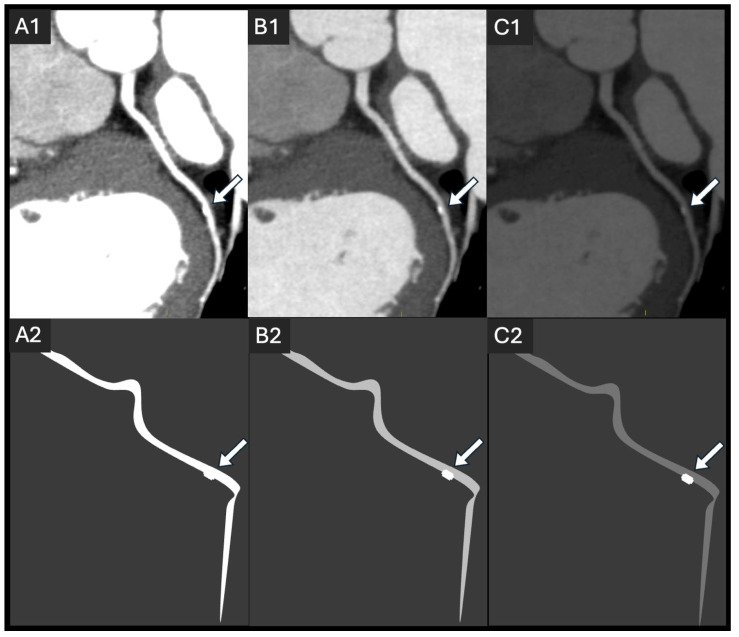
A simulation of the effect of lumen attenuation differences in a 54-year-old patient (**A1**–**C1**) with corresponding schematic representations (**A2**–**C2**). The image shows a single small, calcified plaque in the circumflex artery (arrow), using different display windows, simulating hyperattenuated (**A1**,**A2**), optimal (**B1**,**B2**), and hypoattenuated (**C1**,**C2**) lumen. A hyperattenuated lumen impairs calcification detection, while a hypoattenuated lumen affects visual lumen analysis.

To achieve optimal coronary lumen enhancement, intravascular access with an 18-gauge IV catheter is recommended; in smaller patients, a 20-gauge catheter can be sufficient [10]. Rapid flow rates (4–7 mL/s) are typically applied, with a total volume depending on the CM iodine concentration, patient weight, and CT system. While the guidelines do not provide clear guidance regarding the CM volume, typical values range from 50 to 130 mL [27]. Research assessing the ideal intraluminal Hounsfield unit (HU) CT number has been conducted for over a decade. The results indicate that both excessively low and excessively high attenuation can have detrimental effects on CCTA interpretation. Vascular attenuation below 300 HU on CCTA leads to limited diagnostic accuracy in detecting coronary artery stenosis, especially in more distal segments [28], and is, therefore, not recommended. On the other hand, CT numbers exceeding 500 HU led to a significant underestimation of stenosis in non-calcified plaque, resulting from blooming artifacts [29] (Figure 1).

However, the development of CT hardware and software and reconstruction algorithms, particularly those based on deep learning, could lead to improvements in contrast and blooming artifact reduction.

**Figure 2:**
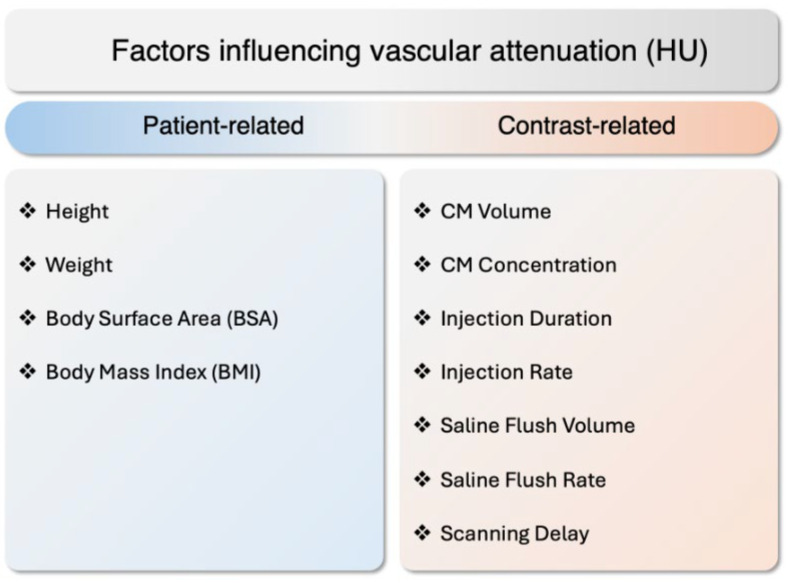
Factors influencing vascular attenuation, separated into contrast-related factors and patient-related factors. CM: contrast media, HU: Hounsfield unit.

Controlling CT numbers [HU] in the coronary lumen has been challenging since vascular attenuation depends on many interacting variables. Anthropometric variables, including height, weight, body surface area, and BMI, are inversely correlated with coronary artery CT numbers [HU] [30]. Furthermore, vascular attenuation also directly depends on the properties of the CM injection. Specifically, the total CM volume, iodine concentration, and injection rate together determine the iodine delivery rate (IDR), which is critical for achieving optimal vascular enhancement. The IDR is calculated as follows: IDR = Iodine concentration [mg I/mL] × injection flow rate [mL/s]. It indicates how fast iodine is delivered into the bloodstream, thereby influencing the peak opacification in the coronary arteries. A higher CM volume or concentration, or a higher injection rate, will increase the IDR and consequently enhance the intraluminal CT numbers [HU] [31] (Figure 2).

In addition, other parameters, such as cardiac output, injection duration, the volume and rate of the saline flush, and the timing of the scanning delay, further influence the behavior of the contrast bolus. Cardiac output affects how rapidly blood, and thus the contrast, circulates through the coronary arteries. The saline flush helps the contrast media bolus progress through the venous system, improving bolus homogeneity and reducing artifacts. Collectively, these factors interact in complex ways to determine the final CT numbers [HU] observed in the coronary lumen, underscoring the necessity of precise parameter optimization to achieve high-quality diagnostic images.

## 3. Current State of Research on Radiation Dose and Contrast Dose

### 3.1. Radiation Dose and Risks

Ionizing radiation carries potential risks [32]. The primary concern is the stochastic effect, particularly the potential for radiation-induced cancer [33]. The long-term risks associated with ionizing radiation exposure are generally low in single CCTA examinations, but they become more relevant when repeated exams are performed due to the cumulative dose. A recent review paper reported typical dose-length products (DLPs) for CCTA ranging from 110 to 904 mGy × cm, suggesting high variability but also relevant progress in saving doses [34]. Large registries (e.g., CRESCENT, PROTECTION, and the German Cardiac CT Registry) have benchmarked radiation doses in clinical practice, highlighting the impact of optimization strategies, such as tube voltage reduction, ECG-based modulation, and iterative reconstruction [35].

CCTA used to be associated with a high radiation dose and has been discussed extensively in the literature. The risks associated with ionizing radiation are of two types: deterministic (tissue reactions) and stochastic (carcinogenic). Deterministic risks occur only when the exposure lies beyond the threshold of 0.1–1 Gy (for a fetus prior to organ formation, this threshold varies between 50 mGy and 300 mGy), which is not reached in medical imaging. On the other hand, stochastic effects occur at whatever the dose and mainly involve genetic changes that can increase the likelihood, not the severity, of malignancy [34]. While some studies have suggested a potential increase in cancer risk associated with radiation doses from CCTA [36], the level of risk is often considered limited and placed in the context of the clinical benefits of the diagnostic information obtained. However, a recent major meta-analysis found an inordinate increase in malignancy risk associated with CT examinations [37]. This emphasizes ongoing efforts to minimize the radiation dose following the “as low as reasonably achievable (ALARA)” principle without compromising diagnostic image quality in CCTA [38]. The trend is moving in the right direction, and some reports even achieved sub-millisievert CCTA, a major step in mitigating radiation risks [39]. Similarly, in dynamic myocardial CT perfusion, reducing the sampling rate has been shown to lower effective doses significantly while maintaining diagnostic accuracy [40]. In clinical practice, such dose levels still seem futuristic. A recent review summarized the radiation dose from cardiovascular CT over 13 years (2007–2020) and reported that DLPs ranged from 171 to 661 mGy.cm, corresponding to an effective dose of 4.5–17.2 mSv, using a conversion factor of 0.026 mSv/mGy×cm [34].

Patient age plays a significant role in susceptibility to ionizing radiation. The risks of radiation-induced stochastic effects are notably higher in children, estimated to be up to four times greater than in adults. While not detailed in this review, specific optimization techniques and recommendations apply to children [41,42]. Conversely, senior patients have a significantly lower risk, reduced by a factor of ten or more compared to younger individuals [43]. This is primarily due to aging-related factors, including reduced cell proliferation and repair, accumulation of cellular damage leading to decreased cellular responsiveness to radiation, and a shorter lifespan, limiting the time for cancer to develop [44]. Age is a critical factor in CCTA, as middle-aged to older adults are the primary population, while younger patients, who more frequently undergo CT scans for congenital heart disease, highlight the need for age-adapted CCTA protocols. Obese patients generally require higher X-ray doses for CCTA. The reason relates to their larger body size and increased tissue density, leading to increased photon attenuation and scatter, which can degrade image quality. To compensate for this, higher tube currents and voltages are often used to ensure adequate image quality and diagnostic accuracy [45].

### 3.2. Iodinated Contrast Volume and Risks

Iodinated CM, delivered intravenously during CCTA, is irreplaceable for visualizing coronary arteries and aids in detecting stenosis and other cardiac abnormalities, and non-contrast cardiac CT value is limited to the coronary artery calcium score. However, the use of iodinated contrast material is not without risks. One concern is the potential for contrast-associated acute kidney injury (CA-AKI), especially in patients with impaired renal function [46]. CA-AKI is characterized by a temporary decline in kidney function following the administration of contrast material (usually within 48–72 h). Patients with pre-existing renal dysfunction, diabetes, dehydration, or a history of contrast allergies are particularly susceptible to CA-AKI. Therefore, carefully considering renal function and appropriate hydration protocols is crucial when using iodinated contrast materials in CCTA [47].

Although the incidence of adverse events has decreased since the advent of non-ionic and low-osmolar CM, risks such as CA-AKI and, at times, life-threatening allergic reactions, persist [48]. Furthermore, a study published in 2019, using a pre-clinical experiment involving minipigs, investigated the contributing effect of CM iodine volume on radiation-induced DNA damage in blood lymphocytes during a CCTA [49]. They also confirmed that a lower CM iodine volume results in a reduced level of DNA damage at a constant radiation exposure. Building upon these findings, the same research group conducted a prospective clinical study published in 2021, which confirmed that the iodine dose in contrast media directly influences the occurrence of radiation-induced DNA double-strand breaks in peripheral blood lymphocytes, with a linear dose-dependent relationship observed in CCTA [50].

Reducing the use of CM material not only improves patient safety but also saves costs, and addresses concerns about the impact of iodine on drinking and surface water quality and supply chain shortages. In fact, the environmental impact of iodinated CM is an emerging concern because the CM is not metabolized and is excreted in the wastewater. It can accumulate in drinking and surface water, raising the question of ecological effects, including toxicity to aquatic life and future water purification challenges [51]. Additionally, the increasing global demand for iodinated contrast agents has highlighted supply chain vulnerabilities, further emphasizing the need to optimize iodine use in medical imaging to reduce both environmental and resource-related pressures [52,53].

## 4. Radiation Dose and Contrast Media Control Approaches

Several factors can be optimized to improve radiation dose and contrast agent volume control in imaging procedures. Key manipulable parameters include reducing the tube voltage, applying prospective ECG-gating techniques, choosing the iodine concentration, volume, and flow rate, and heart rate control medication. These variables can be adjusted to improve image quality while minimizing radiation exposure to the patient [54]. Both current and emerging techniques (see Section 4) are summarized in Table 1.

### 4.1. Tube Kilovoltage Peak (kVp)

Variations in tube kilovoltage peak (kVp) can significantly contribute to CM volume control in coronary CT angiography (CCTA). Low tube voltage protocols, such as 80 kVp or 100 kVp, combined with prospective ECG-triggering, have been shown to reduce the radiation dose by up to 38% while maintaining good image quality [23,55]. Recent studies have demonstrated that 80-kVp protocols paired with DLR can achieve even greater reductions in both the radiation dose and contrast volume, compared to 120-kVp protocols with iterative reconstruction, while simultaneously improving signal-to-noise and contrast-to-noise ratios, even in overweight patients [56,57]. Reducing the tube voltage in CT angiography offers an additional benefit as it enhances the intravascular contrast medium attenuation, resulting in an improved contrast-to-noise ratio [58]. However, low tube voltage usage can also influence plaque morphology. Specifically, it has been shown to increase luminal CT numbers [HU], which may result in an apparent increase in calcified plaque volume and a corresponding reduction in fibrofatty and necrotic core components. These findings are important to consider, particularly as CCTA-based plaque measures are increasingly used to guide medical management and evaluate treatment response [59].

**Figure 3:**
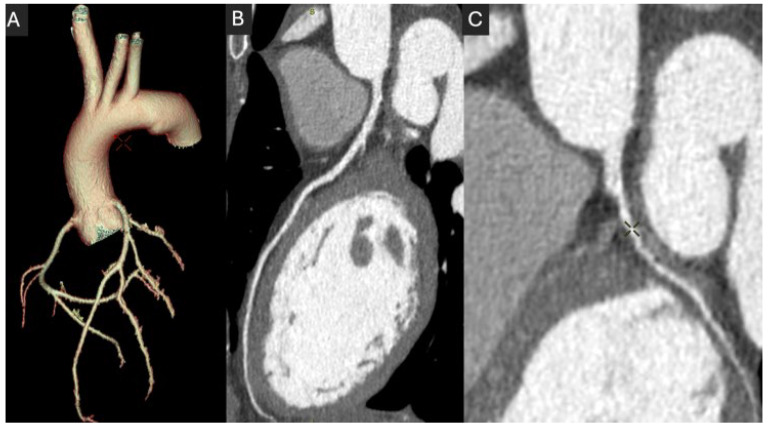
An example of a thoracic CTA in a 23-year-old woman with suspected Ehlers–Danlos syndrome, presenting with exercise-related chest pain. The scan was performed with a low-dose protocol (80 kV, 460 mA, and 0.23 s rotation), resulting in an effective dose of 1.13 mSv. The aorta and coronary arteries are visualized in a 3D volume-rendered reconstruction (**A**), with a full curved MPR at 90° rotation (**B**), and a zoomed-in curved MPR at 0° rotation (**C**), focusing on the left anterior descending artery (LAD). The image quality was optimal, allowing us to rule out both coronary and aortic disease.

Figure 3 shows a CCTA of a 23-year-old woman in which, given the patient’s young age, the protocol was optimized to reduce the dose delivered while maintaining high-quality imaging, as follows: 80 kV, 460 mA at a high rotation speed of 0.23 s. It resulted in a CT dose index (CTDI) of 4.4 mGy and DLP of 87 mGy.cm, corresponding to an effective dose of 1.13 mSv, using a 0.013 mSv·mGy^−1^·cm^−1^ conversion factor. For reference, the 2024 UK diagnostic reference levels for chest CT angiography are 9.1 mGy CTDI and 310 mGy.cm DLP [60].

### 4.2. Prospective ECG-Gating

Prospective ECG-gating refers to a technique where the timing of the R-wave is predicted before the scan, enabling image acquisition during a defined window in the cardiac cycle, typically in diastole, for minimizing motion artifacts in standard coronary evaluation, while systolic phase imaging may be valuable for assessing dynamic phenomena, such as coronary systolic bending, intramural functional occlusion, or myocardial bridging. This approach is opposed to retrospective gating, which uses continuous scanning throughout the entire cardiac cycle. Notably, prospective gating can reduce the radiation dose significantly, with up to a 77–85% reduction compared to retrospective gating, while maintaining similar image quality [61,62]. While effective in patients with stable, low-bpm cardiac rhythms, prospective ECG-gating can be more challenging in patients with irregular or fast heart rates, where accurate timing and image acquisition may be compromised [63].

### 4.3. Iodine Injection Protocol

In CCTA, the effectiveness of the CM injection depends on several key parameters, including the CM concentration, injection volume, flow rate, and, ultimately, the IDR. The CM concentration refers to the amount of iodine per milliliter of contrast agent (e.g., 300–400 mg I/mL), influencing the contrast enhancement achieved. The injection volume, usually ranging from 50 to 130 mL, is the total amount of contrast administered during the scan. The flow rate, usually between 4 and 7 mL/s, controls how quickly the contrast is delivered. Ultimately, the key factor is the IDR (see Section 1). Typical IDR values for CCTA range from 1.5 to 2.0 gI/s, which ensures an optimal vascular enhancement, though DLR-enabled protocols can maintain diagnostic quality even at 0.5 mL/kg contrast volumes [64].

Adjustments to these parameters can significantly impact both the radiation dose and CM volume. For example, lowering the tube voltage from 100 kVp to 80 kVp achieved a 16% reduction in effective dose, while increasing the iodine concentration from 350 mgI/mL to 400 mgI/mL and raising the noise index from 25 to 30 levels resulted in an additional 26% dose reduction [65]. Conversely, some studies have explored the effects of lowering iodine concentration. By combining reduced tube voltage and iterative reconstruction techniques, the use of a lower iodine concentration contrast agent (270 mg I/mL) achieved a 26.5% reduction in iodine volume and a 34.9% reduction in effective dose, while maintaining comparable image quality and attenuation levels [66].

**Figure 4:**
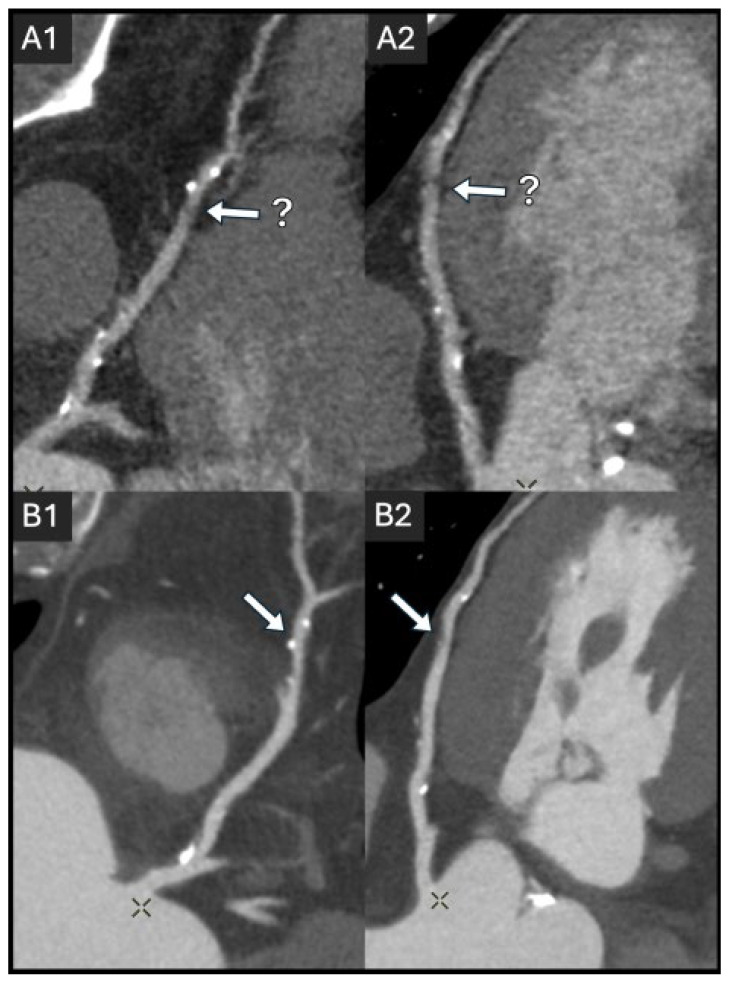
An example of the effect of suboptimal iodine delivery in a 63-year-old patient with endocarditis and severe aortic stenosis, undergoing coronary CT angiography (CCTA) to rule out coronary stenosis. The initial scan, performed at 87 bpm via an 18 G peripherally inserted central catheter (PICC), resulted in a limited maximum flow rate (50 mL, approximately 3 mL/s). Due to insufficient lumen attenuation and limited diagnostic accuracy (**A1**,**A2**), this scan did not allow for ruling out a stenosis of 50% or more, with a plaque of undetermined diameter stenosis (white arrow with a question mark). While the PICC model was designed to handle up to 5 mL/s flow, several factors can influence whether this flow is achievable, including the catheter length, contrast medium (CM) viscosity, and injection pressure. A repeat CCTA, following proper venous access (18 G IV catheter in the antecubital fossa) and beta-blocker preparation (72 bpm), achieved optimal image quality using 50 mL of CM at 5 mL/s with motion correction (**B1**,**B2**) and showed a <50% stenosis (white arrow). The images are displayed in a curved MPR view at 0° and 90° rotation, respectively. The CT protocol was exactly the same for both acquisitions: tube voltage, 100 kVp; tube current, 900 mA; and gantry revolution time, 0.23 s. The difference in noise between (**A1**,**A2**) and (**B1**,**B2**) is due to the lower heart rate in the repeat scan. At a lower heart rate, more projections (or data samples) contribute to the reconstruction of a single slice, resulting in lower noise despite identical acquisition parameters.

Figure 4 illustrates the impact of non-optimal vs. optimal iodine injection protocols to obtain diagnostic-quality images.

Despite a generally accepted consensus aiming to reduce the use of CM in cardiovascular CT, a meta-analysis highlighted significant variations in acquisition techniques, patient characteristics, and CM injection parameters, making it challenging to establish standardized recommendations [18]. For instance, there is a lack of consensus on the optimal combination of CM concentration, IDR, flow rate, total volume, or timing for patients with varying body weights, heart rates, and cardiac outputs. Typically reported injection protocols advocate for high iodine concentrations (320–400 mg/mL), volumes ranging from 50 to 100 (even 130) mL, and flow rates of at least 4 mL/s [31]. Similar to radiation dose, iodine contrast protocols should be tailored to individual patient characteristics to balance diagnostic efficacy with the risks of contrast-associated adverse events and optimize outcomes. In particular, patients with a greater body mass index typically require a higher CM dose volume; however, obese individuals have a substantial proportion of fatty tissue that barely influences arterial enhancement. Based on this fact, studies have demonstrated that CM volume based on lean body weight allows for optimization, helping to reduce the CM volume administered in CT angiography for patients with a BMI > 25 kg/m^2^ [67].

Recently, the “double low” strategy has gained attention for its ability to simultaneously reduce both the radiation dose and iodinated CM burden. This approach combines low tube voltage protocols with a reduced CM volume administration, achieving substantial reductions in both radiation exposure and the risk of contrast-induced nephropathy, without compromising diagnostic image quality [68].

### 4.4. Heart Rate Control

While advancements in CT technology have made it possible to reduce scan time and radiation dose, heart rate remains a significant factor influencing image quality and dose. A lower-range physiologic (60–65 bpm) and stable heart rate allows for better image quality with reduced motion artifacts, which is particularly important for coronary artery assessment. To achieve this, β-blockers are commonly used to lower the heart rate before the scan [39]. Administered orally or intravenously, β-blockers effectively bring heart rates down to around 65 beats per minute (bpm), thereby facilitating optimal imaging and thus avoiding the need for retrospective acquisition. Additionally, beta-blockers can be administered to patients with a pacemaker if the heart rate is higher than the paced rhythm threshold and if there are no pacing spikes visible on the ECG. Once the spikes are visible, indicating that the device is pacing, the heart rate cannot be reduced with medication [69]. Although the use of β-blockers is considered safe, it may be associated with side effects, necessitating a thorough review of the patient’s medical history to identify any contraindications, such as severe asthma, severe chronic obstructive pulmonary disease, heart block, or decompensated heart failure [70].

## 5. Emerging Technologies and Techniques

The ongoing evolution of CT technology has led to the development of several promising advancements designed to further optimize radiation dose, reduce CM volume, and improve image quality. These innovations include advancements in gantry rotation speeds, detector size, dual-source technology, iterative reconstruction, and, more recently, DLR [16] and AI-driven CCTA analysis [71]. Dual-source CT systems have demonstrated consistent advantages over single-source scanners in clinical practice, with studies reporting both lower radiation doses (~3.7 vs. 4.8 mSv) and superior coronary artery delineation when using prospective gating [72]. Finally, the advent of PCCT has emerged as the ultimate milestone in cardiac imaging, allowing for radiation dose and CM reduction, while enhancing stenosis quantification and qualitative image quality [73].

### 5.1. Gantry Rotation

Gantry rotation refers to the movement of the CT scanner’s X-ray tube and detectors around the patient during image acquisition. The typical range of gantry rotation speeds in modern CT scanners generally falls between 0.25 and 1.0 s per rotation. For high-speed scans, such as those used in cardiac imaging, gantry rotation times of around 0.35 s or faster are common [74]. The speed of gantry rotation plays a crucial role in determining the scan’s quality and efficiency since it is directly correlated with temporal resolution. Faster gantry rotation speeds allow for quicker acquisition times, reducing the duration of patient exposure to radiation [75]. Faster gantry rotation speeds are recommended for coronary CT angiography (CCTA), as they not only impact radiation dose but also enhance image quality by reducing motion artifacts, thereby improving the overall diagnostic clarity. Additionally, this speed increase has the potential to allow CCTA to be performed in patients without the need for oral beta blockers, simplifying the procedure and reducing patient preparation time [76].

### 5.2. Detector Size

Recent advancements in CT scanner technology have led to significant changes in detector size. While 64-detector CT scanners were once the largest detector coverage in clinical practice, the introduction of 128-, 256-row, or even 320-row scanners has become increasingly available in modern imaging [77]. These detectors offer several advantages, including enhanced spatial and temporal resolution, as well as improved image quality due to greater coverage. Specifically, a 256- or 320-row scanner allows for heart imaging in a single rotation, significantly reducing scan times and completely avoiding misregistration artifacts. This directly impacts the radiation dose, with a reduction in radiation exposure by at least 20% compared to 64-row scanners, because larger arrays cover a larger portion of the heart in a single rotation and require fewer rotations to sample the whole heart [78]. However, their diffusion is limited worldwide due to the high cost and the prevalent cardiac imaging use.

### 5.3. Iterative and Deep-Learning Image Reconstruction

Iterative reconstruction techniques have shown considerable advancement in radiation dose reduction in CCTA by reducing image noise without compromising image quality [79]. The use of iterative reconstruction can lead to a substantial reduction in the effective dose of radiation, with reductions of up to 48% compared with filtered back projection (FBP), showing either equal or improved image quality, in terms of noise, contrast-to-noise ratio, and subjective image quality [15,80,81].

Deep learning-based reconstruction (DLR) is emerging as a novel algorithm able to achieve FBP image quality while significantly reducing radiation dose [82]. DLR introduces a more advanced approach, using artificial intelligence to enhance image clarity, reduce noise, and minimize artifacts while preserving spatial resolution [16]. Compared to iterative reconstruction algorithms, DLR reduces noise while maintaining noise texture, with a slight improvement in spatial resolution. This leads to a greater potential for radiation dose reduction compared to IR techniques. In fact, DLR has shown the potential to provide up to a 64% dose reduction in a phantom study, using quantitative model observer analysis [83]. A recent DL-based noise-reduction method achieved 64.5% lower noise and 2.9× higher CNR without compromising CAD-RADS accuracy [84]. Its development continues to evolve, and it holds great promise for safer and more efficient CT imaging.

### 5.4. Spectral CT and Photon Counting CT

In recent years, dual-energy CT (DECT) systems have become widely available, offering spectral capabilities that can benefit patients in different ways. Because iodine has a high probability of photoelectric effect near its K-edge, approximately 33.2 keV, DECT can be used to exploit this absorption to enhance CT numbers [HU] in the arterial lumen by applying lower energy (40–60 keV range) virtual monochromatic imaging. Therefore, using low keV reconstruction enables the potential to reduce the volume of the CM injected into the patient while maintaining image quality similar to that of a single-energy CT. PCCT, a more recent and sophisticated spectral CT technology, has already demonstrated the benefits of low keV reconstruction (50 keV) in a phantom study, showing higher iodine CT numbers [HU] and improved contrast-to-noise ratio [85]. For instance, DECT has been shown to allow for a 40% CM volume reduction in detector-based DECT for coronary artery imaging while maintaining image quality and even enhancing the contrast-to-noise ratio [86]. The more recent introduction of PCCT represents a transformative advancement in computed tomography. Unlike traditional energy-integrating detectors, PCCT directly measures individual X-ray photon energies, offering improved spatial resolution, reduced image noise, and enhanced spectral imaging capabilities. This novel technology addresses many limitations of conventional CT by enabling better soft tissue differentiation, reduced artifacts, and potentially lower radiation doses, making it a promising tool for clinical imaging and research [87,88]. PCCT has shown promise in addressing the limitations of conventional CCTA, such as spatial resolution and blooming artifacts. Ultra-high-resolution reconstructions with PCCT enable precise plaque characterization and better in-stent lumen assessment, even in heavily calcified vessels [89,90,91].

The potential of dose reduction in coronary artery CT with PCCT has yet to be thoroughly investigated. The high spatial resolution and noise-reducing capabilities allow for high-quality imaging at lower radiation levels. A recent study demonstrated that high-pitch PCCT can achieve diagnostic coronary imaging with as little as 30 mL contrast medium while improving stenosis assessment confidence through multi-energy reconstructions [92]. Additionally, PCCT’s ability to perform monoenergetic imaging and spectral reconstruction helps minimize iodine load without sacrificing diagnostic accuracy [93,94,95].

## 6. Recommendations for Practitioners

**Figure 5:**
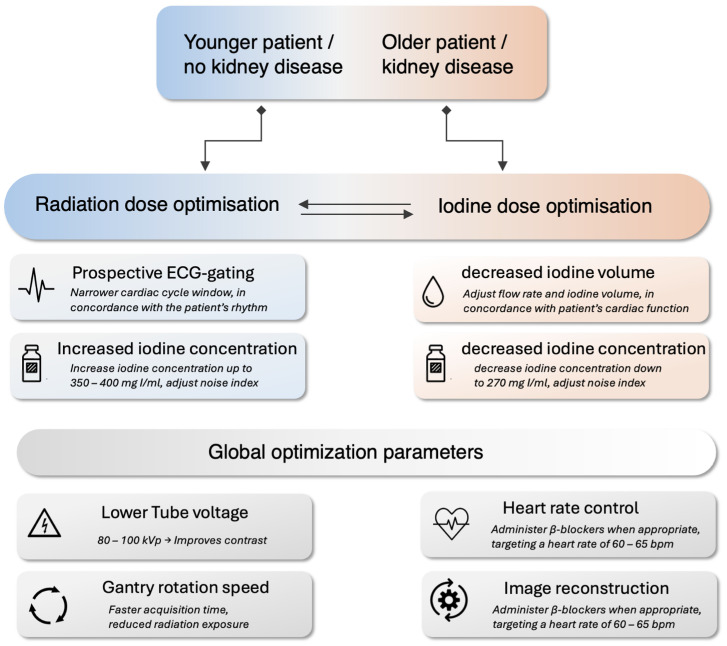
Suggestions for practitioners regarding radiation dose and iodine dose optimization based on patient characteristics, as well as general optimization parameters. Note that although obese patients are not specifically mentioned in this algorithm, they require both higher X-ray and contrast medium doses. Reducing either dose can compromise the clinical usefulness of CCTA in this patient group.

Determining CCTA protocols, balancing radiation dose and iodinated CM, requires a personalized approach that prioritizes diagnostic accuracy while minimizing risks. We propose a practical framework for cardiac imagers to achieve this compromise (Figure 5), focusing on CT system optimization and patient-specific protocol optimization, including strategies to prioritize ionizing radiation and/or iodine dose reduction based on patient characteristics. Physicians in charge should always follow the “as low as reasonably achievable” (ALARA) principle for radiation dose, particularly in younger patients more susceptible to ionizing radiation’s long-term stochastic effects. However, applying the ALARA principle to iodine load helps reduce the risk of contrast-induced nephrotoxicity, healthcare costs, supply chain pressure, radiation-induced DNA damage, and environmental impact.

### 6.1. CT System and Protocol Optimization

Modern CT systems offer a wide range of hardware and software features that can be adjusted to reduce both radiation and iodine doses without sacrificing image quality. Wide-area detectors or dual-source systems are recommended to reduce scan times and radiation exposure [94].

Acquisition parameters should be optimized, including reduced tube voltage (e.g., 80–100 kVp) and maximum rotation speed [10]. Modern reconstruction algorithms can help compensate for the increased noise. Use prospective ECG-gating whenever possible, limiting the acquisition windows to the required cardiac phase (s) only, significantly reducing radiation dose compared to retrospective gating.

Implement DLR or iterative reconstruction algorithms to reduce noise and improve image quality, enabling lower radiation doses (usually lower tube current). Use motion correction algorithms to minimize artifacts, particularly in patients with higher heart rates, mitigating the need to repeat acquisition.

### 6.2. Patient-Specific Protocol Optimization

Patient-specific optimization requires actively thinking about both radiation and iodine dose based on individual characteristics, as each patient presents unique challenges.

Younger patients, as their organs are more radiosensitive, benefit the most from radiation dose reduction strategies, such as a lower tube voltage (80–100 kVp), while maintaining a high iodine concentration (350–400 mg I/mL) and at least a 4 mL/s flow rate to optimize vascular attenuation [65]. Because the risk of nephrotoxicity and cardiovascular load from iodine injection is lower in these patients, the CM administration can be traded off to reduce the radiation dose further.

Older patients or those with kidney disease who are at increased risk of acute kidney injury, especially those with cardiovascular disease, require iodine dose reduction strategies [96]. These may include lowering the iodine concentration and adjusting the injection parameters to maintain image quality while minimizing the risk of nephrotoxicity. In such cases, the tube voltage should be kept as low as possible to optimize the intraluminal CT number [HU], and it may be useful to increase the tube current to improve the contrast resolution, compensating for the lack of contrast, and resulting in a higher radiation dose. Each case must be considered individually, with careful attention to the risk–benefit balance when making adjustments to these parameters.

Heart rate control remains a key factor in both image quality and dose efficiency and should be aimed at for all patients, if possible. Administering β-blockers, when appropriate, to achieve a controlled heart rate reduces motion artifacts and allows for better coronary visualization with minimal radiation exposure.

Obese patients represent a unique patient group, posing the particular challenge of requiring higher X-ray dose to compensate for their high tissue attenuation, and needing more iodinated CM to account for the increased blood volume and fight qualitative image quality loss due to noise. However, the increase in CM volume is not proportional to the weight and should be optimized individually [67].

## 7. Conclusions

Coronary CT angiography (CCTA) has undergone rapid technological advancements, leading to significant improvements in image quality, diagnostic accuracy, and patient safety. In this review, we explored the key technical parameters influencing CCTA performance and how they impact image quality and diagnostic utility. Among these, radiation dose and iodine contrast dose remain critical and often competing factors that must be balanced carefully. The optimal trade-off between the two is not universal but should be tailored to the individual characteristics of each patient, including age, cardiovascular risk, and renal function.

Recognizing this complexity, we provide summarized, practical guidelines to support care providers in selecting the most appropriate CCTA protocols. These recommendations are designed to help achieve diagnostic-quality images while minimizing risks, thereby promoting safer and more effective patient care.

## Figures and Tables

**Table 1:** Summary of current and emerging dose control techniques.

	Description	Key Benefits
Current Techniques		
Tube Voltage (kVp)	Lowering the tube voltage to 80 kVp (70 kVp in children) or 100 kVp.	Reduces radiation dose and enhances contrast-to-noise ratio.
Prospective ECG-gating	A technique where image acquisition is synchronized with the R-wave, enabling a defined window in the cardiac cycle.	Significant reduction in radiation dose compared to retrospective gating.
Iodine Concentration	Adjusting iodine concentration and/or flow rate.	Increasing iodine concentration or flow rate enhances image contrast, thereby improving lesion detectability. Can reduce radiation exposure and effective dose. Conversely, lowering it can reduce iodine volume with minimal quality loss.
Heart Rate Control	Use of β-blockers to lower heart rate.	Reduces motion artifacts, improves image quality, and minimizes radiation exposure thanks to shorter exposure time and fewer repeat scans.
**Emerging Techniques**		
Gantry Rotation Speed	Faster gantry rotation speeds.	Faster acquisition times, reduced radiation exposure, and reduced motion artifacts.
Detector Size	Larger detector sizes (e.g., 256 or 320 rows).	Enhance spatial and temporal resolution and reduce radiation exposure.
Iterative Reconstruction	Enable reduction in pixel noise standard deviation.	Enable a reduction in mA during image acquisition, thereby lowering the required radiation dose and maintaining similar image quality compared to filtered back-projection (FBP).
Deep-Learning Reconstruction (DLR)	Uses artificial intelligence to reduce noise, improving image quality.	This algorithm leads to a decrease in radiation dose by decreasing mA during acquisition while maintaining a similar noise texture to FBP.
Photon-Counting CT (PCCT)	A new technology that directly measures individual X-ray photon energies.	High spatial resolution, reduced noise, lower radiation doses, improved soft tissue differentiation, and better plaque characterization in CCTA.

## Data Availability

No new data were created.

## Abbreviations

The following abbreviations are used in this manuscript:

BMI	Body mass index
BPM	Beats per minute
CA-AKI	Contrast-associated acute kidney injury
CAD	Coronary artery disease
CCTA	Coronary computed tomography angiography
CM	Contrast medium
CTDI	Computed tomography dose index
DLP	Dose-length product
DLR	Deep learning-based reconstruction
FBP	Filtered back projection
HU	Hounsfield unit
IDR	Iodine delivery rate
PCCT	Photon-counting computed tomography
